# Effect of band-aligned double absorber layers on photovoltaic characteristics of chemical bath deposited PbS/CdS thin film solar cells

**DOI:** 10.1038/srep14353

**Published:** 2015-09-23

**Authors:** Deuk Ho Yeon, Bhaskar Chandra Mohanty, Seung Min Lee, Yong Soo Cho

**Affiliations:** 1 Department of Materials Science and Engineering, Yonsei University, Seoul 120-749, Korea; 2 School of Physics & Materials Science, Thapar University, Patiala, 147004, India

## Abstract

Here we report the highest energy conversion efficiency and good stability of PbS thin film-based depleted heterojunction solar cells, not involving PbS quantum dots. The PbS thin films were grown by the low cost chemical bath deposition (CBD) process at relatively low temperatures. Compared to the quantum dot solar cells which require critical and multistep complex procedures for surface passivation, the present approach, leveraging the facile modulation of the optoelectronic properties of the PbS films by the CBD process, offers a simpler route for optimization of PbS-based solar cells. Through an architectural modification, wherein two band-aligned junctions are stacked without any intervening layers, an enhancement of conversion efficiency by as much as 30% from 3.10 to 4.03% facilitated by absorption of a wider range of solar spectrum has been obtained. As an added advantage of the low band gap PbS stacked over a wide gap PbS, the devices show stability over a period of 10 days.

In recent years, solution-processed inorganic thin film photovoltaics have exhibited tremendous potential in the quest for sustainable energy sources^1^,^2^,^3^,^4^. Among the various materials that have been aggressively pursued for the purpose, PbS has emerged as one of the top candidates over the last decade^3^,^4^. Although photoresponse of PbS has been long revealed, the recent development is based on its quantum dots that provide with ability to exploit the near infrared (NIR) region due to band gap tunability as a consequence of quantum size effects^5^. Significant and rapid progress has been made on solar cells based on the solution-processed quantum dots from less than 1% efficiency in 2005 to more than 8% in 2014^6^,^7^,^8^.

Till date, architectures of (i) Schottky junction between a quantum dot film and metal electrode^9^, (ii) depleted heterojunction where a quantum dot film is sandwiched between a metal and an n-type conductor like TiO_2_^2^, ZnO^10^ or CdS^4^ and (iii) quantum dot-sensitized solar cells^11^ have been employed for photovoltaic applications. In a true sense, however, the quantum dot thin film is a misnomer; it is rather a two dimensionally constricted assembly of individually surface-passivated quantum dots. Incidentally, surface chemistry of the quantum dots is one of the factors those critically affect the energy conversion efficiency^12^,^13^. It has been accepted that the solution-processed quantum dots exhibit poor air stability that can pose serious apprehensions on fabrication of scalable low cost devices^9^,^14^. In turn, this has prompted researchers to explore other possibilities that can yield high performance at a reasonably low cost. For example, recently it has been suggested to use pulsed laser ablation-based synthesis of the PbS quantum dots^15^. Some other research group has used elemental sulfur, albeit multiple processing steps, instead of the conventional bis (trimethylsilyl) sulfide owing to its pyrophoric nature and high cost^16^.

In this work, we seek to address the need for development of PbS absorber layers via a cost competitive solution process that offers the advantageous feature of band gap tuning similar to that of the quantum dots, without involving multi-step chemical processing and/or without ligands engineering. Our approach is based on fabrication of thin PbS layers by the chemical bath deposition (CBD) method wherein the band gap can be tailored by the process parameters such as precursor concentration, temperature, etc. For example, the processing temperature controls the crystallite size of the films, which determines the optical band gap of PbS^17^,^18^,^19^. A higher bath temperature was reported to induce a larger crystallite size by precipitating the nucleation and growth process of PbS and, thus, a lower band gap with potential changes in unfilled inter-granular volume and localized energy states^17^. While the quantum dot approach has drawn significant attention, there have been only a few attempts in preparing CBD PbS based solar cells. Hernandez-Borja *et al.*^18^ had reported CBD PbS/CdS thin film solar cells with an efficiency of 1.63%. Using a microwave-assisted CBD process, a similar efficiency of 1.68% for the PbS/CdS solar cells was achieved by Obaid *et al.*^19^ In this communication, a novel concept of stacking of absorber layers grown by the CBD process has been examined as a pathway for improved performance. Built on the strategy of multijunctions, we provide the evidences that a graded recombination layer, as proposed recently^20^,^21^, is not necessary between two p-type PbS layers of different band gaps, if the band alignment is suitably engineered.

In the multijunction devices, the partner junctions having different band gap absorber materials sequentially harness power from their respective portions of the sun’s broad light spectrum, and hence, the tandem and multijunction devices are expected to fare better than the single junction ones. However, the success of such devices lies in the current-matching of the junctions and efficient recombination of photogenerated electrons and holes from adjacent junctions^20^,^22^. For this purpose, in compound semiconductor multijunction solar cells, a tunnel junction of heavily-doped p and n-type semiconductors has been used^22^,^23^,^24^,^25^, which is extremely difficult to replicate in the solution-based low temperature processes. Recently, for a stack of two depleted heterojunctions of PbS quantum dots, multiple intervening layers have been used with the sole objective of the sandwiched layers being to allow the photo-electrons of the back junction to recombine with the photo-holes of the front junction^20^,^21^,^26^. For the first time, herein, we show that indeed two band align engineered junctions can be stacked directly to produce an efficiency higher than their individual ones. This approach is benefited from the facile modulation of the otpo-electronic properties of the PbS thin films using the CBD process. The results are quite promising with a conversion efficiency of 4.03 %, when considered the non-colloidal approach without involvements of quantum dots.

## Experimental Procedure

Commercially available fluorine-doped tin oxide (FTO)-coated glass substrates (Pilkinton, U.K.) were cleaned in an ultrasonic bath for 10 min each with acetone and deionized water, and then dried with nitrogen gas. For n-type semiconductors, CdS thin films were grown by the CBD process using a solution containing 100 mL deionized water, 0.025 M cadmium nitrate (Cd(NO_3_)_2_∙4H_2_O) (98%, Kanto Chemical Co., Ltd, Tokyo, Japan), 0.15 M sodium citrate (Na_3_C_6_H_5_O_7_) (99%, Duksan, Gyungkido, Korea), 0.3 M ammonia (NH_3_) (25%, Duksan, Gyungkido, Korea), and 0.05 M thiourea (CH_4_N_2_S) (99%, Aldrich, Milwaukee, WI). The cleaned FTO glass substrates were vertically immersed into the precursor solution at 80 °C for 20 min. This process was repeated twice to obtain pinhole-free CdS films with a thickness of ~50 nm.

The PbS thin films were deposited on the CdS-coated FTO/Glass substrates by the CBD process. A 100 ml of aqueous solution containing 0.05 M lead nitrate (Pb(NO_3_)_2_) (99.3% Kanto Chemical Co., Ltd, Tokyo, Japan), 0.04 M triethanolamine (C_6_H_15_NO_3_) (99%, Aldrich, Milwaukee, WI), 0.2 M sodium hydroxide (NaOH) (95%, Duksan, Gyungkido, Korea) and 0.06 M thiourea was prepared. The PbS films with a band gap of 1.61 eV were deposited by dipping the substrates into the precursor solution at 40 ^o^C for 60 min to obtain a 100 nm thick film. In order to obtain the PbS film (E_g _= 0.92 eV) of varied thicknesses, the deposition was carried out at a bath temperature of 80 °C for different durations ranging from 1 to 20 min. The crystal size of the 40 and 80 °C-processed samples were 16 and 52 nm, respectively^17^. After each deposition (for CdS and PbS), the samples were cleaned in an ultrasonic bath with deionized water followed by drying in nitrogen gas. An Al electrode with a thickness of 100 nm was deposited by thermal evaporation at a vacuum of 1 × 10^−6^ Torr to complete the solar cell devices.

Optical transmittance and reflectance of the films were measured using a UV-visible spectrophotometer (V530, JASCO). Surface and cross-sectional microstructures was observed by field emission scanning electron microscopy (FESEM: JSM-7001F, JEOL). Ultraviolet photoelectron spectroscopy (UPS: PHI 5000 VersaProbe^TM^, ULVAC-PHI) was used to obtain electron binding energy spectra. Hall measurement was carried out at room temperature using a Hall measurement system (Ecopia 21, HMS-3000, Korea).

Solar cells based on the structure of Al/PbS(0.92 eV)/PbS(1.61 eV)/CdS/FTO/glass with a cell size of 0.09 cm^2^ were fabricated. Devices using a single absorber layer either of 1.61 or 0.92 eV band gap were also prepared. Current density-voltage (J–V) measurements were obtained using an I–V curve analyzer (IviumStat, Ivium Technology) and a solar simulator (Sun2000, ABET technology) at AM 1.5. The external quantum efficiency (EQE) of the cells was analyzed by using an incident photon conversion efficiency measurement unit (QEX10, PV measurements).

## Results and Discussion

**Figure 1:**
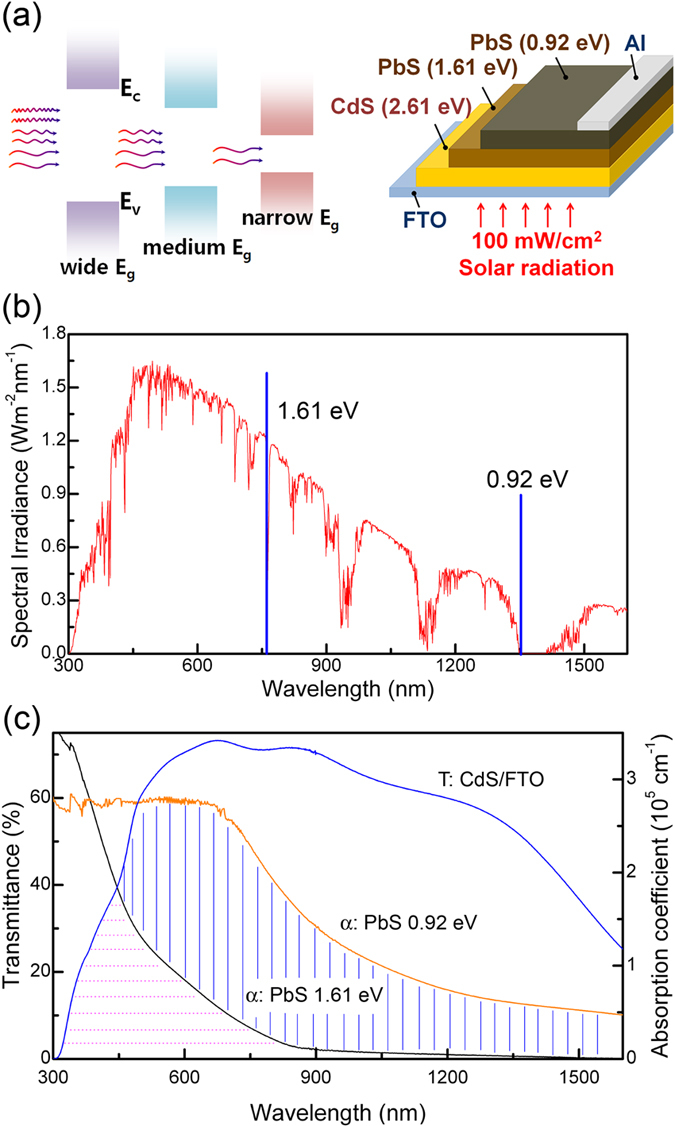
(**a**) Stacking of PbS layers of different band gaps allows absorption of a wider range of solar spectrum. The wider band gap layer absorbs short wavelength photons while the longer wavelength photons are transmitted through it to reach the narrow band gap PbS. The schematic of the approach is also given. **(b**) Proposed utilization scheme of solar spectrum. (**c**) Typical transmittance of the CdS/FTO/Glass and absorption coefficient of PbS thin films with band gaps of 0.92 and 1.61 eV. Stacking of 0.92 eV band gap PbS thin film improves absorption range of solar spectrum.

Our approach towards the enhancement of PbS based solar cells is illustrated in Fig. 1. The PbS layers of band gaps of 1.61 eV and 0.92 eV are stacked directly: the wider band gap layer forming the front junction. A thin CBD n-type CdS layer was used as the electron extracting layer following the favorable results in the PbS quantum dot-based solar cells^16^. The n-p junction reportedly facilitates efficient collection of carriers from the built-in electric field created near the junction, typical of depleted heterojunction architectures. Upon irradiation, a significant portion of sun light, mainly of short wavelength photons, is absorbed in the large band gap PbS and the relatively longer wavelength photons reach to the narrow band gap PbS/Al junction. This is reflected from the absorbance spectra of the individual layers, as presented in Fig. 1(c). Devices with single layers yielded the conversion efficiencies of about 3.10% for 1.61 eV band gap PbS and 0.19% for 0.92 eV band gap PbS^17^. An UPS study revealed that there exists a barrier for electron injection from the 0.92 eV PbS layer to the CdS layer (Supplementary Figure S1 and Figure S2). The difficulty in overcoming this barrier for the photogenerated electrons is primarily the reason for the poor performance of the PbS (0.92 eV)/CdS junctions. Accordingly, the concept of double layers as described above is expected to boost the performance.

The overall efficiency of the devices critically depends on quenching of the exciton recombination and efficient extraction of the carriers. This means that the photogenerated electrons in the 1.61 eV band gap PbS layers must be readily injected to the CdS layer, and be collected at the electrode therefrom. This is ensured by limiting the thickness of the PbS (1.61 eV) layer such that it is fully depleted. It also requires that the n-type CdS layer must be thin (~50 nm) because of the low carrier lifetime. Furthermore, the depleted heterojunction provides with an effective barrier to hole injection into the electron-extracting electrode. Assuming that this devices work optimally, still a large portion of photon energy (<1.61 eV) is left uncapped. We propose to replace the top half of the 1.61 eV band gap PbS layer by another PbS layer of lower band gap (Fig. 1a), so that it can be photoexcited by the long wavelength photons and if these excitons can be harnessed to the existing carrier currents due to the 1.61 eV band gap PbS, this device architecture can be highly beneficial. In the process, the total thickness of the absorber layer remains invariant (~200 nm).

**Figure 2:**
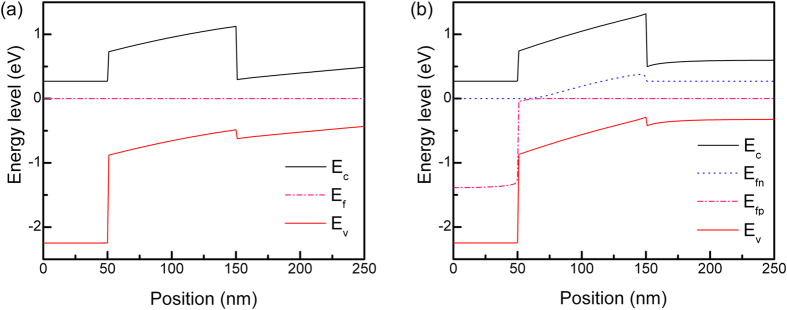
Energy level diagrams of the devices with stacked PbS layers under (**a**) **dark and** (**b**) **illuminated conditions.** E_fn_ and E_fp_ denote the quasi-Fermi levels of electrons and holes, respectively.

We synthesized n-type CdS layer of about 50 nm thickness that had a work function (ϕ) of 3.74 eV, an electron affinity (χ) of 3.47 eV and a band gap (E_g_) of 2.61 eV (Supplementary Figure S3). The wider band gap PbS thin films had ϕ = 4.34 eV and χ = 3.11 eV while the corresponding values of the 0.92 eV band gap PbS film were 4.43 and 3.94 eV, respectively^17^. It indicates that the energy difference between the vacuum level and the top of the valence band decreased with the increase in the band gap (i.e., a difference of 4.86 eV for the 0.92 eV band gap PbS layer vs 4.72 eV for the 1.61 eV band gap PbS layer). This trend, however, is opposite to that observed for the PbS quantum dots having diameter in the range of 3–10 nm^27^. This difference may be due to the fact that the strong quantum confinement effect in the quantum dots that results in increased band gap is not operational in the present case due to the larger crystallite size. Figure 2 presents band diagram of the structure under dark and 1.5 AM illumination, as simulated by PC1D^28^ using the results of the Hall and UPS measurement (Table 1 and Supplementary Table S1). As revealed from the figure, the wider band gap PbS layer being only 100 nm thick is depleted entirely and the energy difference between the front (in contact with CdS) and back (in junction with 0.92 eV PbS) was as high as 0.5 eV. Additionally, starting from the front (PbS/CdS) junction, splitting of the electron and hole quasi-Fermi levels gradually widened to the extent of about 0.3 eV under illumination. Overall, this depicts a scenario strongly favored for carrier transport in the devices. The designed back junction is a staggered type II isotype p-p heterojunction, wherein the hole concentration of the layers differ by about two orders of magnitude (Supplementary Table S1). The splitting of the electron and hole quasi-Fermi levels remain constant at about 0.25 eV under illumination, marginally down from that at the back of the wide band gap PbS layer. The very small potential barrier corresponding to about 0.1 eV is not expected to be a hindrance to the hole collection as the top valence band is sloped favorably towards the electrode. It would rather assist in blocking the photohole migration to the 1.61 eV band gap PbS layer from the 0.92 eV band gap PbS film. As a consequence, the concept of stacked PbS layers would yield better performance than the individual ones.

**Figure 3:**
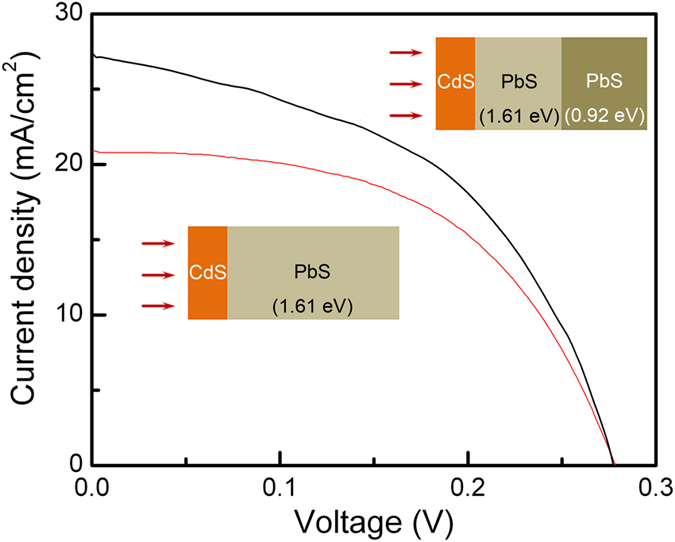
Typical J–V plots of photovoltaic devices with a single 200 nm thick PbS layer (E_g _= 1.61 eV) and of a device with a stacked layer as shown in Fig. 1(a).

The current density (J) - voltage (V) characteristics of the proposed device structure is presented in Fig. 3. The J-V curves at light and dark for an extended bias range from −1 V to 1 V have been provided in the supplementary Figure S4. Compared to a single absorber layer (E_g _= 1.61 eV) of 200 nm thickness, the device (combined thickness of the absorber layers ≈ 200 nm) showed significant improvement as high as 30% in the power conversion efficiency (PCE) from 3.10 to 4.03%. The enhancement was primarily due to the large increment in the short-circuit current density (J_SC_) from about 20.9 to 27.7 mA/cm^2^, assisted by the stacking of the lower band gap PbS that allowed larger portions of the solar spectrum to be absorbed (Fig. 1). This was supported by the results of the external quantum efficiency (EQE) measurement of the devices employing single and stacked absorber layers (Supplementary Figure S5). Compared to the device with a single PbS layer (E_g _= 1.61 eV), the device with stacked absorber layers exhibited enhanced spectral response in the region of wavelength higher than 500 nm, which was primarily responsible for the improved performance of the device.

**Figure 4:**
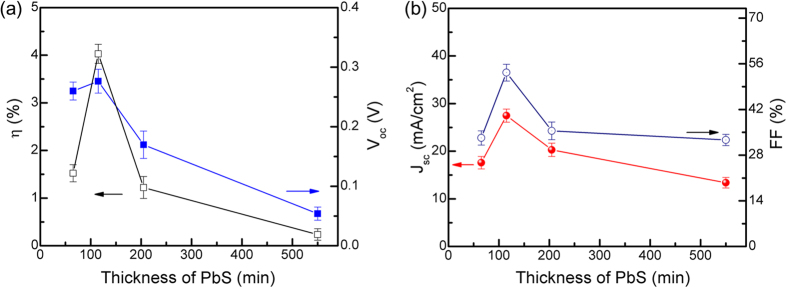
Plots of the variation of the PCE, V_OC_, J_SC_ and FF as a function of thickness of the lower band gap PbS film. The thickness of the wider band gap PbS layer was kept constant at about 100 nm. The uncertainty reflects the spread of the data measured from at least 10 devices.

Figure 4 shows the variation of the PCE, fill factor (FF), J_SC_ and open-circuit voltage (V_OC_) as a function of the lower band gap PbS film thickness. The J_SC_ increases up to a certain optimum thickness of the layer at about 100 nm. This is because at this thickness the layer is fully depleted resulting in the efficient charge extraction by the built-in electric field. As the thickness is increased beyond the optimum value, a portion of the film away from the PbS/PbS junction becomes undepleted by forming a quasi-neutral region. Although owing to higher thickness more light is absorbed in these devices, the photogenerated carriers created in or travelling through this region have higher probability of recombination due to short diffusion lengths of the carriers and hence, contributed less photocurrents. For the optimum thickness of the PbS layer (E_g _= 0.92 eV), a high FF of about 53.3% was observed, whose origin lies in the strong electric field formed across the absorber layers. As the thickness of the layer (E_g _= 0.92 eV) was increased, a quasi-neutral layer was formed in the absorber layer and the overall strength of the electric field is decreased. Also, the formation of a quasi-neutral layer implied that the carriers would move through diffusion rather than drift, which led to an increase in series resistance and decrease of FF. Concurrently, with increase in thickness from the optimum value, significant decrease in the V_OC_ was observed that suggests increasing reverse saturation current density due to high recombination in the quasi-neutral region. On the other hand, for devices with thinner 0.92 eV band gap PbS layer, the performance was poorer, mainly driven by poor microstructure of the film. In the CBD process, the uniform and complete coverage of the substrates by the film proceeds from heterogeneous nucleation followed by crystal growth. As presented in the (Supplementary Figure S6), the 1 min deposition yielded a discontinuous PbS film (E_g _= 0.92 eV) with crystallite diameters of about 55 nm. The device with these discontinuous films showed very poor FF and J_SC_ resulting in inferior PCE.

**Figure 5:**
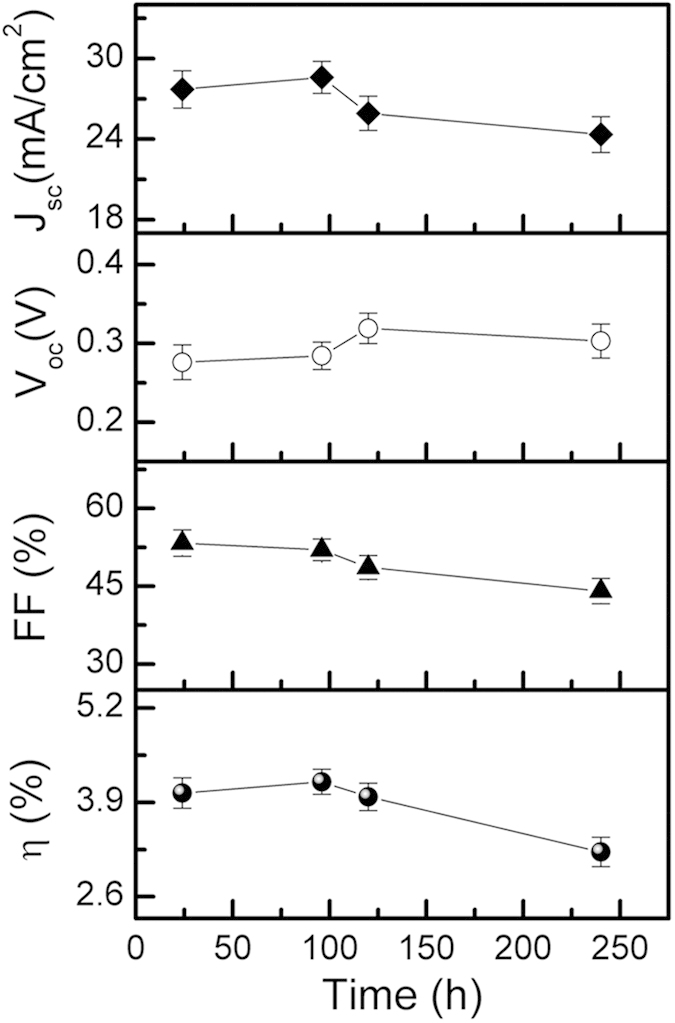
Long-term stability assessment of the devices of stacked absorber layers with the lower band gap PbS film of optimum thickness (~100 nm). The curves are normalized to the performance achieved immediately after the fabrication of the device.

We then explored the additional advantages of the stacking of the layers on air and light stability of the devices. Reported PbS colloidal quantum dot-based solar cells suffer from the lack of stability upon air and light exposure with the possible reasons being loss of passivation of quantum dots under oxygen and moisture exposure, and reaction of metals with ligands in the layer^9^,^14^. Endeavors are on globally to improve the stability that can pave way for commercialization of the PbS based devices. Figure 5 depicts the variation of the device performance as a function of ambient storage time. During the period of assessment of stability, the devices were stored on lab bench without humidity and moisture control, and light shielding. Their photovoltaic characteristics were evaluated periodically in ambient at room temperature without any other precaution such as glove-box and controlled environment^8^. The results indicated superior stability of the devices over a time period as high as 10 days. The PCE was degraded by only about 20% over 250 h of device fabrication and storage. We have also evaluated the stability of a device with a 200 nm thick PbS layer (E_g _= 1.61 eV) which showed a PCE of 3.10%. Strikingly, compared to the device with stacked layers, the single absorber layer device showed considerable degradation in less than 4 h. Since identical layers of CdS and Al were used as electron extraction and contact electrode in both cases, we conclude that the greatly improved stability is due to the stacked low band gap PbS film. One of the possible reasons of improved stability could be its higher χ value of the lower band gap PbS layer compared to that of the underlayer (1.61 eV band gap PbS film). Semiconductors with higher values of χ are thought to be less reactive and hence, less susceptible to performance degrading reactions^29^,^30^.

## Conclusions

This work highlights the potential to improve the PCE of PbS-based solar cell devices through stacking of two band-aligned junctions without any intervening layers. The devices showed an enhancement of PCE by as much as 30% from 3.10 to 4.03% by facilitating absorption of a wider range of solar spectrum, which is a distinct advantage of the modified cell architecture. This approach, which is benefited from the facile modulation of the opto-electronic properties of the PbS thin films using the low cost single step CBD process, offers a simpler route for optimization of PbS-based solar cells. As an added advantage of the low band gap PbS stacked over a wide gap PbS, the devices show good stability over a period of 10 days.

## Additional Information

**How to cite this article**: Ho Yeon, D. *et al.* Effect of band-aligned double absorber layers on photovoltaic characteristics of chemical bath deposited PbS/CdS thin film solar cells. *Sci. Rep.* **5**, 14353; doi: 10.1038/srep14353 (2015).

## Supplementary Material

Supplementary Information

## Figures and Tables

**Table 1:** Parameters used for PC1D simulation.

**Film material**	**Thickness (nm)**	**Dielectric constant**	**Electron affinity**	**Carrier concentration (cm**^−**3**^)	**Fermi level (eV)**
PbS (1.61 eV)	100	43	3.11	5.59 × 10^16^	4.34
PbS (0.92 eV)	100	43	3.94	8.93 × 10^14^	4.43
CdS	50	8.9	3.47	1 × 10^20^	3.74
