# Supplementary material for: Effect of band-aligned double absorber layers on photovoltaic characteristics of chemical bath deposited PbS/CdS thin film solar cells
Source: Sci Rep. 2015 Sep 23;5:14353. doi: 10.1038/srep14353 (PMC4585813; doi:10.1038/srep14353)
Supplement: Supplementary Information [file srep14353-s1.doc]

Supporting Information

Effect of band-aligned double absorber layers on photovoltaic characteristics of chemical bath deposited PbS/CdS thin film solar cells

Deuk Ho Yeon^1,†^, Bhaskar Chandra Mohanty^2,†^, Seung Min Lee^1^, Yong Soo Cho^1^,*

^1^Department of Materials Science and Engineering, Yonsei University, Seoul 120-749, Korea

^2^School of Physics and Materials Science, Thapar University, Patiala, 147004, India

*Corresponding author: [ycho@yonsei.ac.kr](mailto:ycho@yonsei.ac.kr) (Y.S.Cho)

^†^These authors contributed equally.

**I. Device modeling**

The spatial band diagrams and variation of electric field across junctions were simulated using the software PC1D (University of New South Wales). Table 1 in the main text lists the parameters used for the simulations. The parameters were obtained from our Ultraviolet Photoelectron Spectroscopy (UPS) and Hall measurements of the studied samples.

**
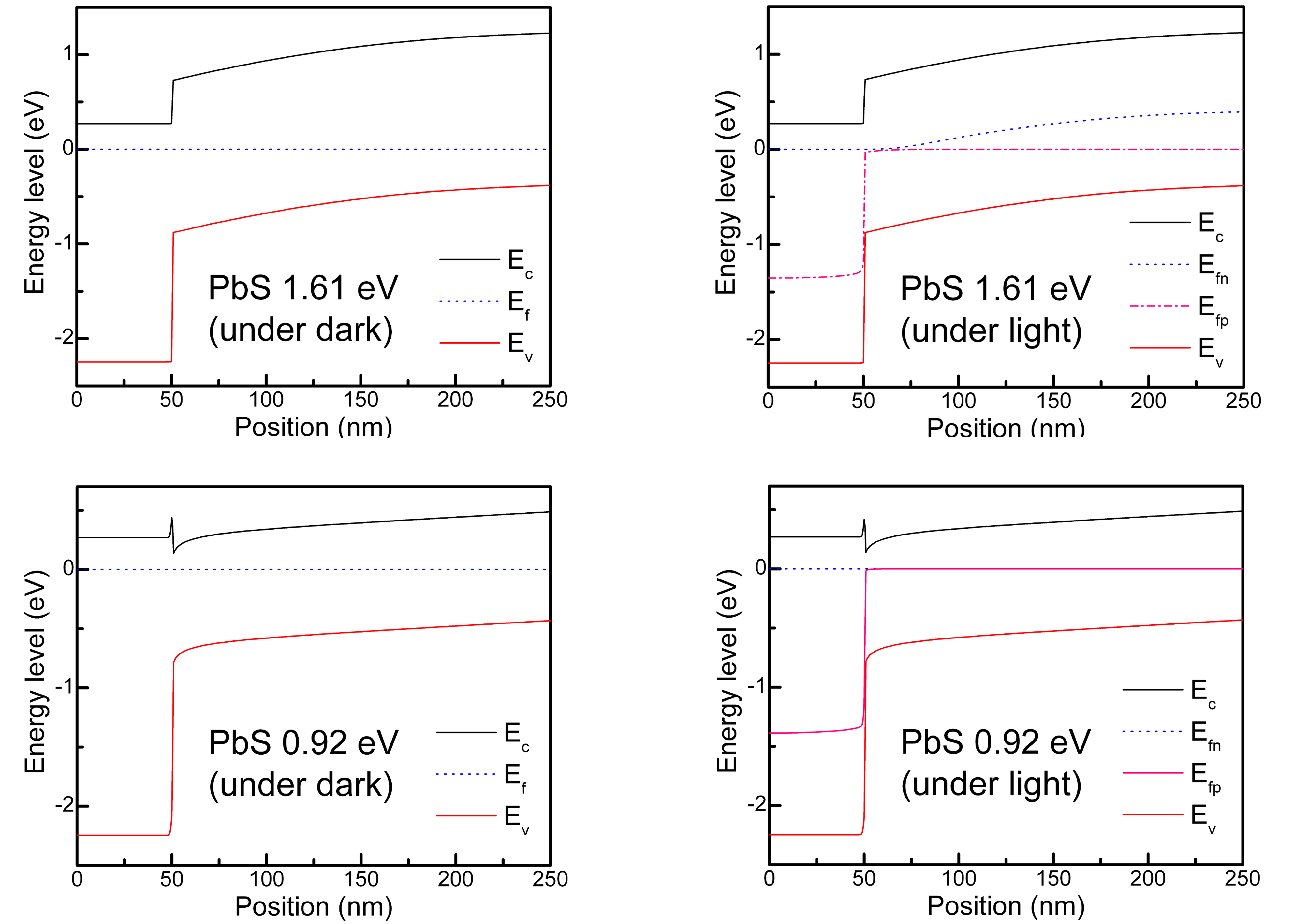
**

**Figure S1:** Simulated band diagrams of CdS/PbS heterojunctions for the PbS films of band gaps of 1.61 and 0.92 eV under dark and illuminated conditions.


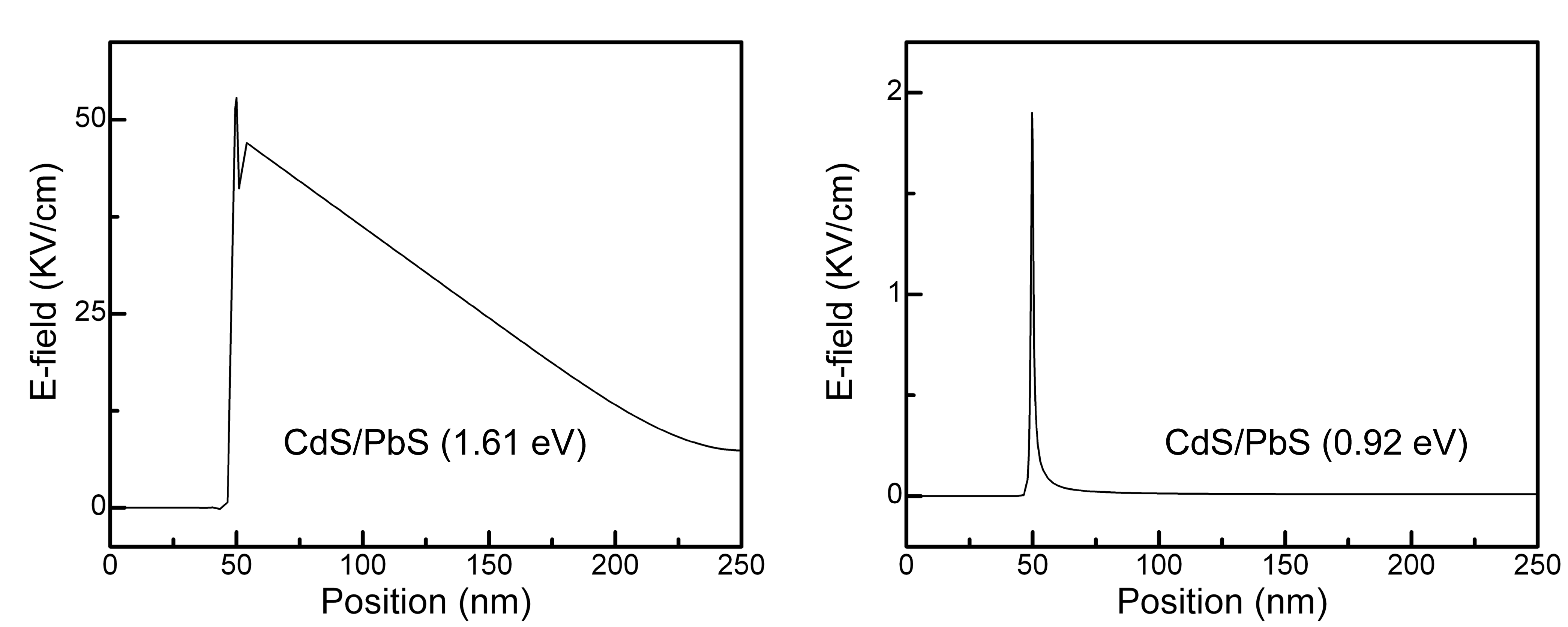


**Figure S2:** Variation of electric field across junctions (a) CdS/PbS (1.61 eV) and (b) CdS/PbS (0.92 eV).

**II. UPS measurement**

The UPS measurements allowed the determination of values of the Fermi level (E_F_) and the top of the valence band position (E_V_). Using the UV-visible-NIR spectroscopy the reflectance and transmittance of the films on glass substrates were measured. Using the reflectance and transmittance data, the optical band gap (E_g_) of the films was estimated. This value of E_g_ was used to locate the bottom of the conduction band of the films.

The UPS was carried out using He I (21.22 eV) photon lines from a discharge lamp.


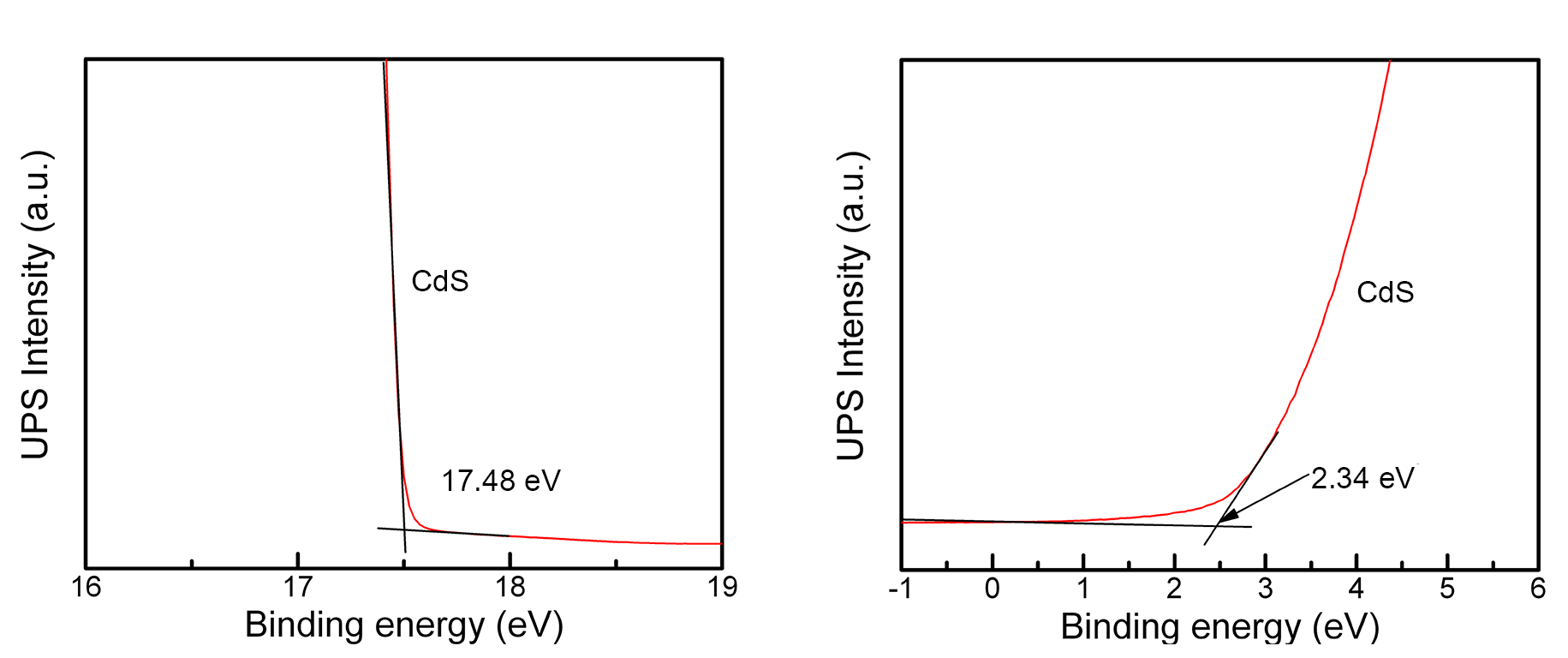


**Figure S3:** Plots of UPS spectra for the CdS films of selected regions. The E_F_ was calculated by subtracting the high-end cutoff value of the curve from the kinetic energy of He I (21.22 eV) photon. The low-end cutoff value of the curve represents the energy below the Fermi level of the material.

**Table S1:** The E_C_, E_V_ and E_F_ values of the materials as determined by the UPS.

| Film material | E_C_ (eV) | E_V_ (eV) | E_F_ (eV) |
| --- | --- | --- | --- |
| PbS  (1.61 eV) | 3.11 | 4.72 | 4.34 |
| PbS  (0.92 eV) | 3.94 | 4.86 | 4.43 |
| CdS | 3.47 | 6.08 | 3.74 |

**III. Current density-voltage (J - V) curves at light and dark for an extended bias range from -1 V to 1 V**


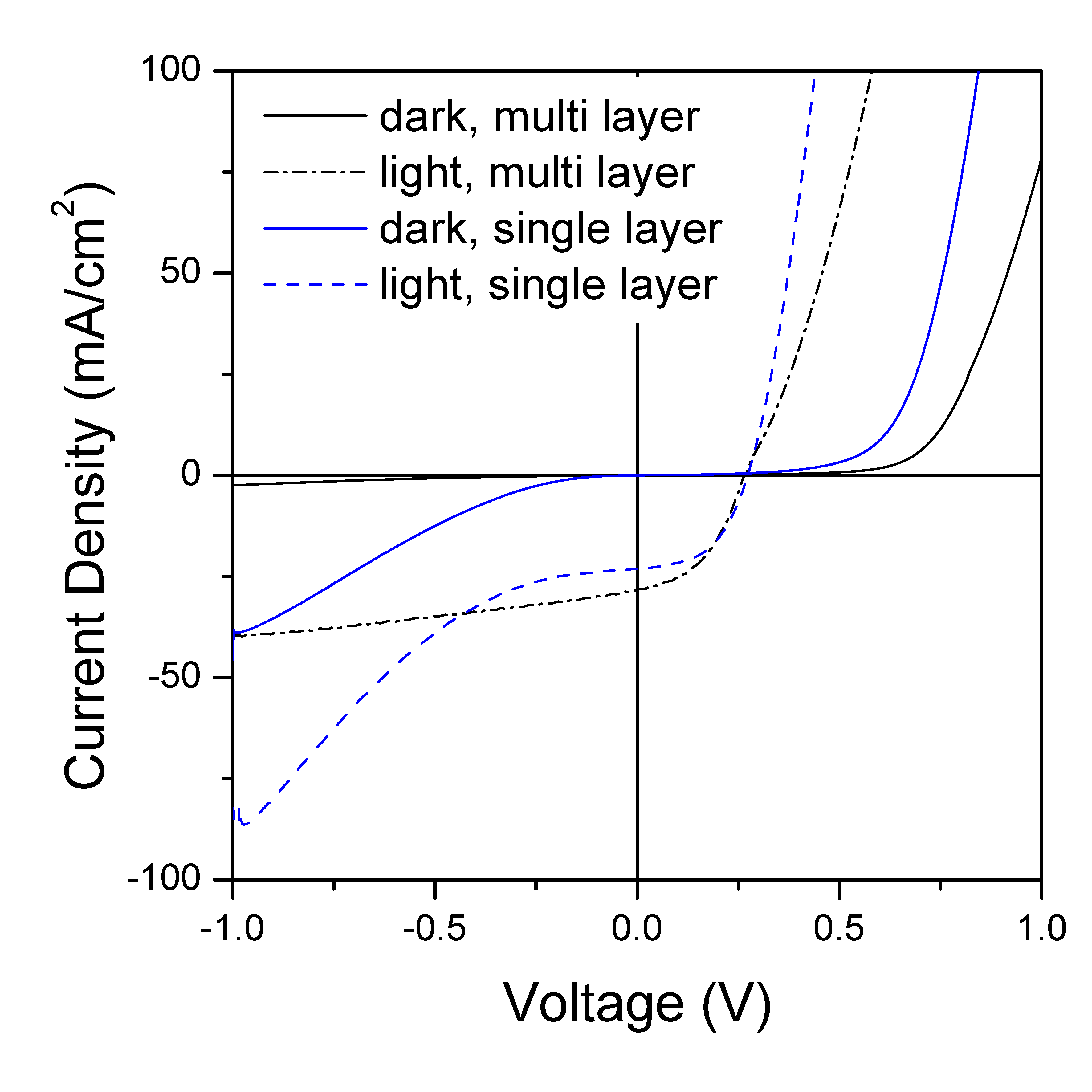


**Figure S4**: J-V curves at light and dark for an extended bias range from -1 V to 1 V for devices with a single PbS (E_g_ = 1.61 eV) absorber layer and multiple (stacked) PbS layers as proposed in Fig. 1.

**IV. Comparison of external quantum efficiency (EQE) of the cells with a single PbS layer (E_g_ = 1.62 eV) and with multiple (stacked) PbS layers as proposed in Fig. 1**


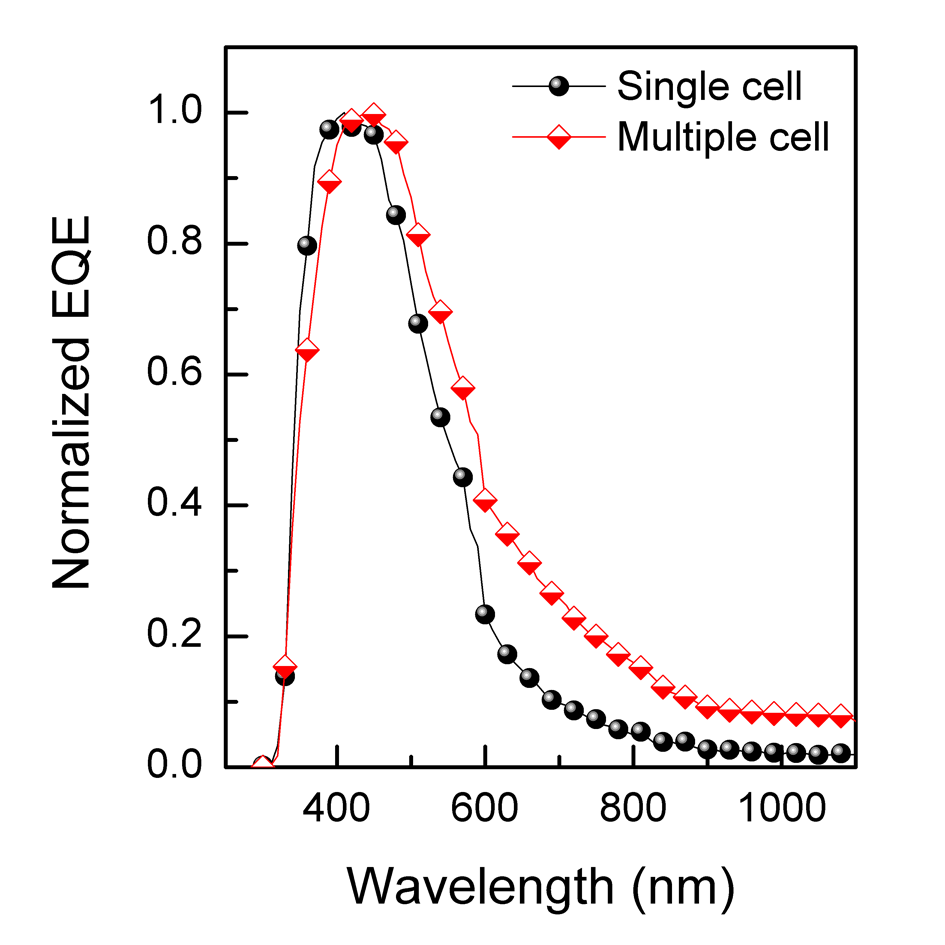


**Figure S5:** The normalized EQE of the devices using a single PbS (E_g_ = 1.61 eV) absorber layer and multiple (stacked) PbS layers as proposed in Fig. 1. As expected, the multiple cell exhibit better spectral response in the region of wavelength higher than 500 nm.

**V. Thickness variation of the PbS film (E_g_ = 0.92 eV)**

**
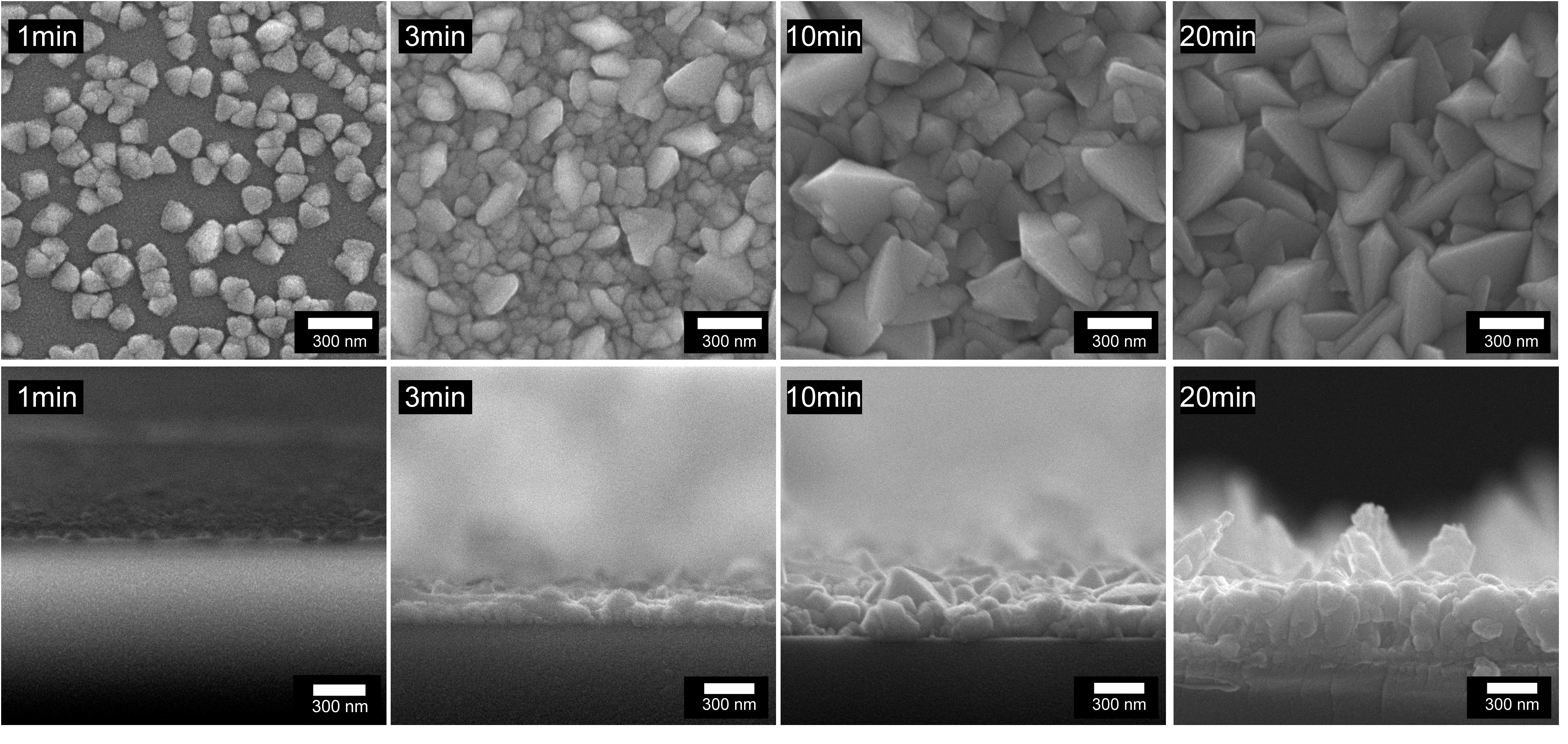
**

**Figure S6:** Surface and cross-sectional SEM images of PbS thin films (E_g_ = 0.92 eV) as a function of deposition time (thickness). The deposition time of 3, 10, and 20 min corresponds to thickness of 115, 205, and 570 nm
